# Is boosting OXPHOS/FAO gene pathways the final end-mechanism of SGLT2i protection?^☆^

**DOI:** 10.1016/j.jmccpl.2025.100297

**Published:** 2025-04-03

**Authors:** Xin Hu, Coert J. Zuurbier

**Affiliations:** Laboratory of Experimental Intensive Care and Anesthesiology, Department of Anesthesiology, Amsterdam Cardiovascular Sciences, Amsterdam UMC, University of Amsterdam, the Netherlands

SGLT2 inhibitors (SGLT2i) have revolutionised medical treatment for a large patient population, *i.e.*, type 2 diabetes, kidney and heart failure patients. Clinical trials with SGLT2i have convincingly demonstrated reductions in hospitalisation for heart failure and progression of kidney disease in patients that were already well-controlled by optimal standard therapy. Thus, patients in the control group already had plasma glucose and lipid levels in an accepted range [1]. In contrast, in many preclinical studies set up to elucidate the underlying mechanisms through which SGLT2i provides such large clinical benefits, the untreated control group often displays unhealthy levels of metabolic systemic parameters, such as hyperglycaemia and hyperlipidaemia. In such studies, SGLT2i-induced protective mechanisms are then convoluted by the often large metabolic systemic effects of SGLT2i, thereby thwarting the interpretation towards the clinical benefits. In the current study, also devised to understand the clinical mechanism providing SGLT2i protection against HF, the authors have used the Gq-overexpressed mice [2]. This is a robust preclinical model well-suited to explore mechanism of HF protection by SGLT2 inhibitors due to the lack of large metabolic systemic disturbances to begin with.

The heterotrimeric G protein Gq is part of a Gq-coupled receptor that is activated by mechanical stressors and/or agonists such as phenylephrine, angiotensin II, endothelin, and prostaglandins to transduce cardiac hypertrophy. Gq activation plays a crucial roles in mediating HF. Cardiac specific overexpression of Gq suffice for inducing adaptive and maladaptive cardiac hypertrophy that is associated with the classic hallmarks of HF: cardiac dysfunction, fibrosis, oxidative stress, and extensive down- regulation of gene pathways associated with mitochondrial oxidative phosphorylation (OXPHOS) and fatty acid oxidation (FAO). The current study demonstrated that treating this genetic model of cardiac hypertrophy/HF with the SGLT2 inhibitor ERTUgliflozin restored almost all of the above mentioned hallmarks of HF [2]. Most remarkably, the gene sets with the most significant coordinate upregulation by ERTU concerned the OXPHOS and FAO pathway. The authors are to be congratulated on bringing together various demanding experimental techniques to allow a broad physiological and genetic characterisation of the Gq-heart failure model along with the treatment effects of the SGLT2i ERTU. Their data strongly indicate that one of SGLT2's primary protective mechanism may well be the improvement of mitochondrial function together with improved FAO. This resonates well with other studies showing that increasing/activating FAO attenuated HF development and improved mitochondrial function [3], possibly due to activation of mitophagy [4].

However, as all good research, important pressing questions remain. The major one is how, molecularly, did ERTU cause this partial restoration of OXPHOS/FAO gene pathways? Recent work has now clearly demonstrated that cardiac protection by SGLT2i is still present in SGLT2-KO animals [5], thus showing that SGLT2i-protection is not through inhibition of the SGLT2 protein, and therefore also not through lowering of plasma glucose or increasing of plasma ketones. One of the earliest findings concerning the underlying mechanisms of SGLT2i is a lowering of intracellular sodium and calcium in cardiomyocytes and endothelial cells through inhibition of the sodium hydrogen exchanger (NHE1) or other plasma sodium loaders [6]. In fact, the authors themselves have already shown that ERTU is associated with lower intracellular sodium in the intact failing heart [7]. Knowing that Gq activation stimulates PLC-β to produce diacylglycerol and IP3 which can then modulate calcium, it is quite possible that ERTU's underlying mechanism of cardiac protection is, at least partly, through the lowering or correction of intracellular calcium levels in the Gq-overexpressed heart. Recognizing that calcium also controls the major cardiac hypertrophy pathway of calcineurin/NFAT, ERTU's changes in OXPHOS/FAO gene expression can then be mediated through the Na/Ca/calcineurin/NFAT pathway (Fig. 1). However, to what extent gene expression of OXPHOS/FAO is controlled by intracellular Na/Ca is a rather an unexplored area, and certainly deserves more attention. Reductions in cytosolic sodium/calcium by ERTU can then also explain the observed reductions in oxidative stress, inflammation, and fibrosis [6]. Thus further research employing the Gq-HF model focussing on the role of plasma sodium loaders and intracellular sodium/calcium in SGLT2i protection is worthwhile to pursue.

**Fig. 1:**
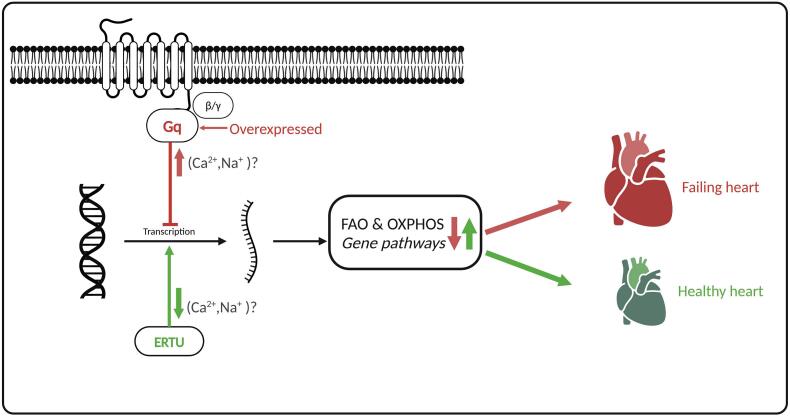
Schematic representation of effects of overexpressed Gq protein and treatment with ERTU on expression of genes related to the mitochondrial oxidative phosphorylation (OXPHOS) and Fatty Acid Oxidation (FAO) and the consequence of these changes on the heart. The red arrows present overexpressed Gq effects, the green arrows ERTU effects. (For interpretation of the references to colour in this figure legend, the reader is referred to the web version of this article.)

Figure created with BioRender.com (licensed to X Hu).

This study, however, is also not without criticism. One major point is the use of ERTU as model for SGLT2i. Despite the fact that many SGLT2i have demonstrated clinical benefits for HF patients, such as for EMPA-, DAPA-, CANA- and SOTAgliflozin, this is not the case for clinically relevant dosages of ERTU. In a preclinical study it has also been demonstrated that at a similar degree of SGLT2-inhibition, a clinically-relevant, low dosage of ERTU was unable to protect the heart, whereas EMPA- and DAPA were protective [8]. Thus, this opens up the question whether the described protective mechanisms of high dosage ERTU in the current work mimic the effects of the clinically-proven SGLT2i. Another critical issue is the rather high dosage of ERTU used in this animal study (500 mg/kg chow). Knowing that ERTU's IC_50_ for SGLT1 inhibition is much lower than that for EMPA (2 μM *versus* 8.3 μM; [9]), the high dosage used likely also inhibits SGLT1 which is present in the heart (in contrast to SGLT2). It can therefore not be excluded that part of ERTU's effects described in current study are due to SGLT1 inhibition. Studying clinically-relevant SGLT2i at clinically-accepted dosage in this model of HF would therefore be important to examine. Finally, for the *ex vivo* analysis in isolated perfused hearts, the hearts were perfused without fatty acids, insulin and lactate and in the absence of ERTU in the perfusate. Thus, these experimental conditions are limiting the potential to understanding ERTUS's effects on *in vivo* cardiac function and metabolism, because of the omission of these important factors that are present in *in vivo* conditions. It is anticipated that ERTU's effects would become even more prominent when these factors are included in the perfusate of the *ex vivo* isolated heart model, recognizing that FAO is increased in ERTU treated animals and that the presence of SGLT2i in the perfusate have direct effects on the metabolism and function of diseased hearts [10].

A blemish does not obscure jade's lustre. Exciting discoveries by Chambers and co-workers suggests that SGLT2i may offer protection through direct restoration of mitochondrial function and fatty acid oxidation. Further research is now needed with clinically-relevant SGLT2i in robust cardiac HF models in the presence of all major metabolic substrates, hormones and the SGLT2i itself, to explore 1) the precise molecular working of SGLT2i effects on OXPHOS/FAO gene pathways, and 2) the causality of improved OXPHOS/FAO for the cardiac protective effects of SGLT2i. Understanding the clinically protective mechanisms of SGLT2i offers an attractive opportunity for the scientific community to facilitate understanding of the major underlying and targetable mechanisms driving human HF.

## CRediT authorship contribution statement

**Xin Hu:** Conceptualization, Writing – original draft, Writing – review & editing. **Coert J. Zuurbier:** Conceptualization, Writing – original draft, Writing – review & editing.

## Declaration of competing interest

The authors declare the following financial interests/personal relationships which may be considered as potential competing interests: Coert J Zuurbier reports a relationship with Boehringer Ingelheim GmbH that includes: funding grants. If there are other authors, they declare that they have no known competing financial interests or personal relationships that could have appeared to influence the work reported in this paper.
